# BAP1, Wilms’ tumor 1, and calretinin in predicting survival and response to first-line chemotherapy in patients with pleural mesothelioma

**DOI:** 10.1007/s00432-023-05565-6

**Published:** 2024-01-27

**Authors:** Tuna Han Yuce, Guntulu Ak, Selma Metintas, Emine Dundar, Oluf Dimitri Roe, Vasiliki Panou, Muzaffer Metintas

**Affiliations:** 1 Department of Chest Diseases, Eskisehir Osmangazi University Medical Faculty, Eskisehir, Turkey; 2 Lung and Pleural Cancers Research and Clinical Center, Eskisehir Osmangazi University, Eskisehir, Turkey; 3 Department of Public Health, Eskisehir Osmangazi University Medical Faculty, Eskisehir, Turkey; 4 Department of Pathology, Eskisehir Osmangazi University Medical Faculty, Eskisehir, Turkey; 5 Department of Oncology, Levanger Hospital, Nord-Trøndelag Hospital Trust, Levanger, Norway; 6 Department of Clinical and Molecular Medicine, Norwegian University of Science and Technology, Trondheim, Norway; 7 Department of Respiratory Medicine, Odense University Hospital, Odense, Denmark; 8 Odense Respiratory Research Unit (ODIN), Department of Clinical Research, University of Southern Denmark, Odense, Denmark; 9 Department of Respiratory Medicine, Aalborg University Hospital, Aalborg, Denmark

**Keywords:** Mesothelioma, BAP1, WT1, Calretinin, Chemotherapy response

## Abstract

**Purpose:**

There are currently no methods to predict response to chemotherapy in pleural mesothelioma (PM). The aim of this study is to investigate the predictive and prognostic role of BAP1, WT1 and calretinin expression and their combinations in pre-treatment tumor samples by immunohistochemical (IHC) staining.

**Methods:**

The study included consecutive PM patients treated with chemotherapy alone at a University hospital between 2009 and 2020. BAP1 analyses were performed on formalin-fixed, paraffin-embedded tumor tissue samples of the patients, while WT1 and calretinin information were obtained from the histopathological diagnosis records.

**Results:**

Of the total 107 patients included, 64% had loss of BAP1 expression, whereas 77% had WT1 and 86% had calretinin expression. Patients with the presence of BAP1 expression, one or both of the other two markers, or loss of expression of all three markers (unfavorable status) were more likely to not respond to chemotherapy than those with the presence of all three markers or loss of BAP1 expression and expression of one or two other markers (favorable status) (*p* = 0.001). Median survival time of patients with favorable and unfavorable status was 15 ± 1.7 and 8.0 ± 2.4 months, respectively (*p* = 0.027). After adjustment for histopathology and stage, loss of BAP1 (HR = 0.54, 95%CI 0.35–0.83), WT1 (1.75, 1.06–2.90), calretinin (2.09, 1.14–3.84) expression and favourable panel (0.50, 0.27–0.92) was associated with prognosis.

**Conclusions:**

The IHC biomarkers BAP1, WT1, and calretinin, used in the routine diagnosis of PM and their combinations, are the first biomarkers associated with response to chemotherapy and may be a useful tool to select patients for first-line platinum pemetrexed treatment in PM patients. Validation in a large cohort is ongoing.

## Introduction

Pleural mesothelioma (PM) is a global health problem and increasing due to its etiopathological relations to asbestos and other carcinogenic mineral fibers. Even four decades after asbestos ban, the incidence is stable in many industrialized countries, and there is unfortunately continued production and use of asbestos, mostly in developing countries (Delgermaa et al. 2011; Ringgaard Petersen et al. 2021; Kindler et al. 2018; Bueno et al. 2018; Røe and Stella 2015). Multimodal treatment, including surgery, radiotherapy, and chemotherapy, contributes to increased life expectancy in suitable patients (Woolhouse et al. 2018; Rimner et al. 2016; Scherpereel et al. 2020). However, since most patients have advanced stage disease at diagnosis, systemic antitumoral therapy remains the main option for treatment. Dual immunotherapy with ipilimumab and nivolumab prolongs survival, mainly in patients with non-epithelioid cell type, compared to the combination of platinum and pemetrexed, which is the standard chemotherapy for PM (Peters et al. 2022). Immunotherapy is expected to be increasingly involved in treating PM in the coming years. However, chemotherapy will continue to be widely used in the treatment of PM for a long time. In both cases, the need for predictive and prognostic tools to determine which patients will benefit from systemic treatment and which patients are expected to have longer survival are imperative to achieve the optimal balance of treatment success, side effects, cost, and patient comfort. Biomarker studies have revealed several candidate biomarkers, but none have made it to clinical practice (Panou et al. 2015) and ongoing clinical trials are also aiming to assess this need in immunotherapy (Fennell et al. 2022). Recently we showed in a validation study of two cohorts that the BRCA1-associated protein 1 (BAP1) expression in tumor was a strong and independent predictor of survival in PM patients treated with first-line pemetrexed-platinum (Louw et al. 2022). Except BAP1, robust prognostic and predictive biomarkers that can determine and follow the course of treatment in PM has not yet been defined.

BAP, Wilms' tumor 1 (WT1), and calretinin immunohistochemical (IHC) staining in tumor tissue are useful markers in the histopathological diagnosis of PM (Chapel et al. 2020). Detection of the BAP1 mutation as a loss of BAP1 protein expression made an important contribution to the differentiation of mesothelioma from benign mesothelial hyperplasia (Cigognetti et al. 2015). WT1 and calretinine expression provide useful decision-making information in diagnosis of PM.

BAP1 is not fully implemented as a clinical prognostic or predictive biomarker yet, mostly due to the lack of large validation studies. In a limited number of studies conducted in recent years, it has been suggested that WT1 and calretinin expression in tumor tissue is associated with a good prognosis in mesothelioma (Farzin et al. 2015; Cedrés et al. 2014; Kao et al. 2011). Since all three markers are almost routinely used in the histopathological diagnosis of PM, if their prognostic and predictive values are determined, they may provide a possibility to select the correct population for chemotherapy and avoid overtreatment.

The aim of this study is to investigate the role of BAP1, WT1, calretinin expression and their combinations in tumor tissue samples by IHC staining in predicting response and prognosis in PM patients treated with chemotherapy alone.

## Materials and methods

### Study design

The study prospectively included consenting patients with histopathological diagnosis of PM at the Department of Chest Diseases, Faculty of Medicine, Eskisehir Osmangazi University and the Lung and Pleural Cancers Research and Clinical Center, 2009–2020. All patients were treated with chemotherapy alone and followed up until death. Data and biological samples were collected for potential research purposes. In this study, BAP1 analyses were performed on formalin-fixed, paraffin-embedded (FFPE) tumor tissue samples of the patients, while WT1 and calretinin information were obtained from the histopathological diagnosis records. The study was approved by the Ethical Committee of Eskisehir Osmangazi University (02.03.2021/19).

The inclusion criteria were age 25–85 years, histopathologically confirmed diagnosis of PM, and treatment with pemetrexed platinum first line. Patients who received palliative radiotherapy for pain control in addition to chemotherapy were also included in the study.

The exclusion criteria were patients with no sufficient tissue samples for IHC staining in tissue blocks or who received best supportive care alone, surgery, or radiation therapy with curative intent for PM or had a history of other active malignancy or cancer treatment.

### Chemotherapy protocols and response measurement

Based on internationally accepted standards in the given time period, pemetrexed-cisplatin or pemetrexed-carboplatin every three weeks were used as first-line therapy. Chemotherapy was discontinued in patients who had progressive disease or did not tolerate the treatment. In patients with stable disease or objective response, five or six cycles were administered. Subsequently, the patients were followed up at appropriate intervals and at pregression treated with either re-induction with pemetrexed platinum or other regimens as gemcitabine or vinorelbine.

The modified Response Evaluation Criteria in Solid Tumor Version 1.1 (mRECIST Version 1.1) was used to evaluate treatment response. Complete response: disappearance of all pleural and non-pleural lesions; partial response: decrease in total tumor measurements of at least 30% compared to baseline; stable disease: decrease of less than 30% or increase in total tumor measurements of less than 20% compared to baseline; progressive disease: increase in total tumor measurements of more than 20% or appearance of a new lesion (Armato and Nowak 2018). Response to chemotherapy was measured every two or three cycles. The response to chemotherapy of patients treated before 2018 was reassessed according to RECIST 1.1.

### Immunohistochemical staining

In the study, hematoxylin and eosin-stained pleural tissue slides obtained from needle biopsy under image (CT or US) guided or medical thoracoscopy were re-evaluated, and the paraffin blocks best representing the morphology were selected for IHC analysis.

#### BAP1

BAP1 immunocytochemistry was applied on 4 μm thick deparaffinized sections using Poly-L-Lysin slides from blocks containing sufficient tissue. IHC staining was performed in accordance with the manufacturer’s instructions. Automatic IHC staining device (Dako Omnis Automated IHC/ISH Staining system) was used in the study. BAP1 polyclonal antibody (BRCA1-associated Protein 1[C4], Catalog Number MC0136, Medaysis) was used at a 1:50 dilution.

#### Calretinin and WT1

Calretinin (Monoclonal Mouse, Clone DAK-Calret 1) and WT1(Monoclonal Mouse, Clone 6F-H2) are two antibodies in the IHC panel routinely used for the diagnosis of mesothelioma at the Pathology Department, along with TTF-1 (Monoclonal Mouse, SPT24) and MOC-31 (Monoclonal Mouse, Clone MOC-31).

#### Evaluation of immunoreactivity

Pancreatic tissue was used as a positive control in IHC analysis with BAP1. Nuclear staining of BAP1 was considered positive (retained) and the absence of nuclear staining was considered as negative (loss of expression). Cytoplasmic staining was not taken into consideration. WT1 and calretinin antibodies were classified as staining positive (expressed) or negative (loss of expression).

### Statistical analyses

Statistical analyses of the study data were performed with the SPSS package program (IBM, version 15.0). Study group data were expressed as central tendency (proportion, mean, rate) and dispersion (standard deviation, min–max). The Chi-square test was used to analyse the qualitative data.

Time between pathologic diagnosis and date of death was considered the overall survival time. Survival was visualized by Kaplan–Meier curves and survival time was expressed as median, mean, and standard error, and 95% confidence interval (95% CI). Log-rank analysis was used to compare survival time between groups.

A Cox regression hazard model was used to determine the variables associated with the probability of survival. Cox regression analysis was performed primarily for univariate variables (age, sex, cell type, stage, response to chemotherapy, IHC staining results of BAP1, WT1, and calretinin), and Hazard Ratio (HR) and 95% CI were calculated. A categorical group was determined for each of the variables, and the HR values of the other groups were calculated according to the categorical group.

In calculating the impact of IHC staining results on survival probability, adjustment was performed by a multivariate Cox regression hazard model for the histopathology and stage. IHC staining results were evaluated both individually and in combination. For this purpose, triple panels were formed based on the staining results, and the relationship between the panel results and response to chemotherapy and prognosis was also examined. The panels were divided into “favourable status” and “unfavourable status” in terms of response to chemotherapy and association with prognosis. An unfavorable status was the loss of expression of all three markers or the loss of one or both of the other two markers in the presence of BAP1 expression. A favorable status was the presence of all three markers or the expression of one or two other markers in the loss of BAP1 expression. A *P* value ≤ 0.05 was deemed significant.

## Results

### Patient characteristics

In total, 107 patients were included in the study (Table 1).

**Table 1:** Clinical characteristics of the patients

Characteristics	Value
Age	
X ± SD (min–max), years	63.83 ± 9.63 (30–81)
Sex, *n* (%)	
Male	68 (63.6)
Female	39 (36.4)
Histopathology, *n* (%)	
Epitheloid	74 (69.1)
Biphasic	25 (23.4)
Sarcomatoid	8 (7.5)
Stage, *n* (%)	
IA	7 (6.5)
IB	11 (10.3)
II	5 (4.7)
IIIA	14 (13.1)
IIIB	55 (51.4)
IV	15 (14.0)
Response to chemotherapy, *n* (%)	
Progressive disease	45 (42.1)
Stable disease	36 (33.6)
Partial response	25 (23.4)
Complete response	1 (0.9)

*SD* Standard deviation

**Fig. 1:**
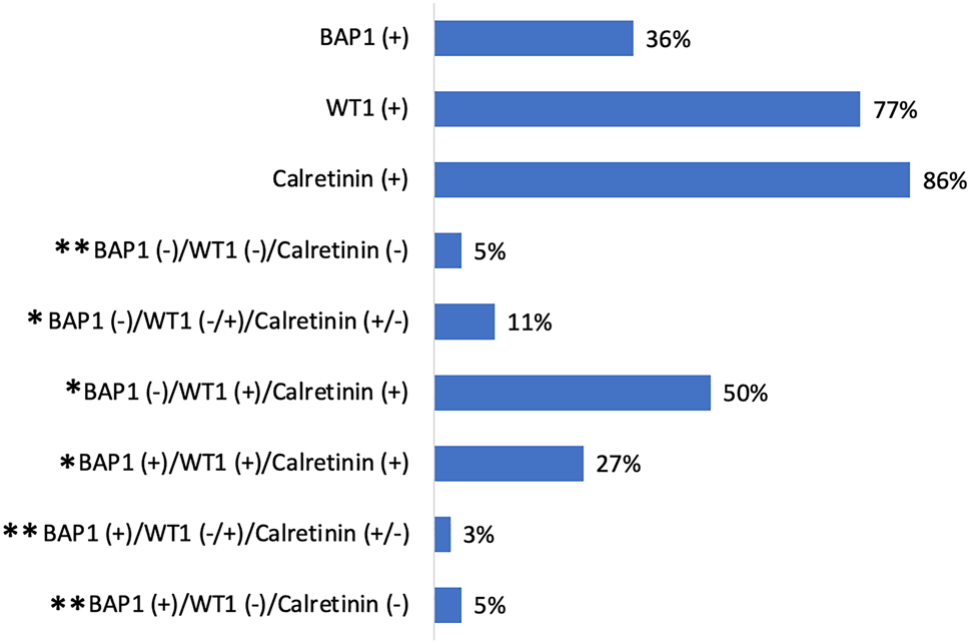
Distribution of immunohistochemical staining in mesothelioma tumor biopsies. BAP1: BRCA1-associated protein 1; WT1: Wilms’ tumor 1; ( +): expressed/retained, (–): loss of expression; (–/ +) and (+ /–): with positive WT1 and negative calretinin or vice versa in the panels. *Favorable; **Unfavorable Missing data: 4

All three IHC stains could be evaluated in 103 patients. Immunohistochemical staining of BAP1, WT1, and calretinin was positive in 36%, 50% and 86%, and panel expressions ranged from 3 to 50% (Fig. 1).

### Immunohistochemical staining, clinical variables and response

Loss of BAP1 expression was more common in female than male (*p* = 0.042). There was no difference in the distribution of BAP1, WT1, and calretinin expression according to patient age and stage. Loss of BAP1 expression did not differ by histopathological type. Expression of WT1 and calretinin was lower in the sarcomatoid type than in the other subtypes (*p* < 0.001 for both). In univariate analysis loss of BAP1 expression was not significantly different between chemotherapy response groups. The expressions of WT1 and calretinin were higher in those with objective response to chemotherapy (*p* = 0.054 and *p* = 0.003, respectively) (see Table 2).

**Table 2:** Distribution of patients’ characteristics and chemotherapy response by immunohistochemical staining results

Characteristics	BAP1 (–)	WTL1 ( +)	Calretinin ( +)
Age, *n* (%)			
< 65	35/49 (71.4)	40/48 (83.3)	44/48 (91.7)
≥ 65	34/58 (58.6)	42/56 (75.0)	48/57 (84.2)
* p*	0.168	0.300	0.248
Sex, *n* (%)			
Male	39/68 (57.4)	51/66 (77.3)	58/66 (87.9)
Female	30/39 (76.9)	31/38 (81.6)	34/39 (87.2)
* p*	**0.042**	0.605	0.916
Histopathological subtype, *n* (%)			
Epithelioid	48/74 (64.9)	62/72 (86.1)	69/74 (93.2)
Biphasic	18/25 (72.0)	19/25 (76.0)	22/25 (88.0)
Sarcomatoid	3/8 (37.5)	1/7 (14.3)	1/6 (16.7)
* p*	0.205	** < 0.001**	< **0.001**
Stage, *n* (%)			
I–IIIA	20/37 (54.1)	28/36 (77.8)	33/37 (89.2)
IIIB–IV	49/70 (70.0)	54/68 (79.4)	59/68 (86.8)
* p*	0.101	0.846	0.719
Chemotherapy response			
Progressive disease	26/45 (57.8)	32/45 (71.1)	33/44 (75.0)
Stable disease	23/36 (63.9)	27/35 (77.1)	33/35 (94.3)
Objective response	20/26 (76.9)	23/24 (95.8)	26/26 (100.0)
* p*	0.266	0.054	**0.003**

Bold *p*-values show statistical significance

**Fig. 2:**
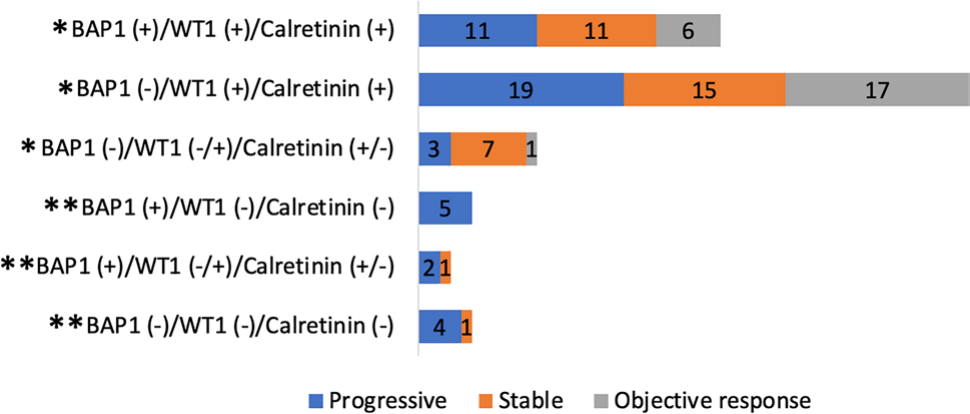
Distribution of response to chemotherapy after the best response according to the immunohistochemical staining combinations. *Favorable; **Unfavorable

The distribution of response to chemotherapy in panels formed according to IHC staining results is in Fig. 2.

An unfavorable status was the loss of expression of all three markers or the loss of one or both of the other two markers in the presence of BAP1 expression. Eleven (84.6%) of 13 patients with unfavorable status had progression. The remaining two patients had a stable disease. Of the 13 patients, 5 had epithelioid, 3 had biphasic, and 5 had sarcomatoid type. A favorable status was the presence of all three markers or the expression of one or two other markers in the loss of BAP1 expression. Twenty-four (26.7%) of 90 patients with favorable status had an objective response to chemotherapy, 33 (36.7%) had stable disease and 33 (36.7%) had progressive disease. Patients with unfavorable IHC status were more likely to not respond to chemotherapy than those with favorable status (*p* = 0.001). After excluding patients with sarcomatoid type, the response to chemotherapy in the remaining 97 patients was worse in the unfavorable status group than in the favorable status group (*p* = 0.046).

### Immunohistochemical staining, clinical variables and survival

One hundred six patients had died at the time of the study (99.1%). The median survival time in the study group was 12.0 ± 1.3 (9.5–1.5) months. Median survival time did not differ by sex (male, female; 11.0, 14.0 months; *p* = 0.259) or age groups (≥ 65, < 65 years; 11.0, 15.0 months; *p* = 0.087). Median survival was 19.0 months in early stage patients (stages I–IIIA) and 10.0 months in advanced stage patients (stages IIIB–IV) (*p* < 0.001). Median survival in patients with epithelioid, biphasic and sarcomatoid cell types was 14.0, 11.0 and 9.0 months, respectively (*p* = 0.076).

**Fig. 3:**
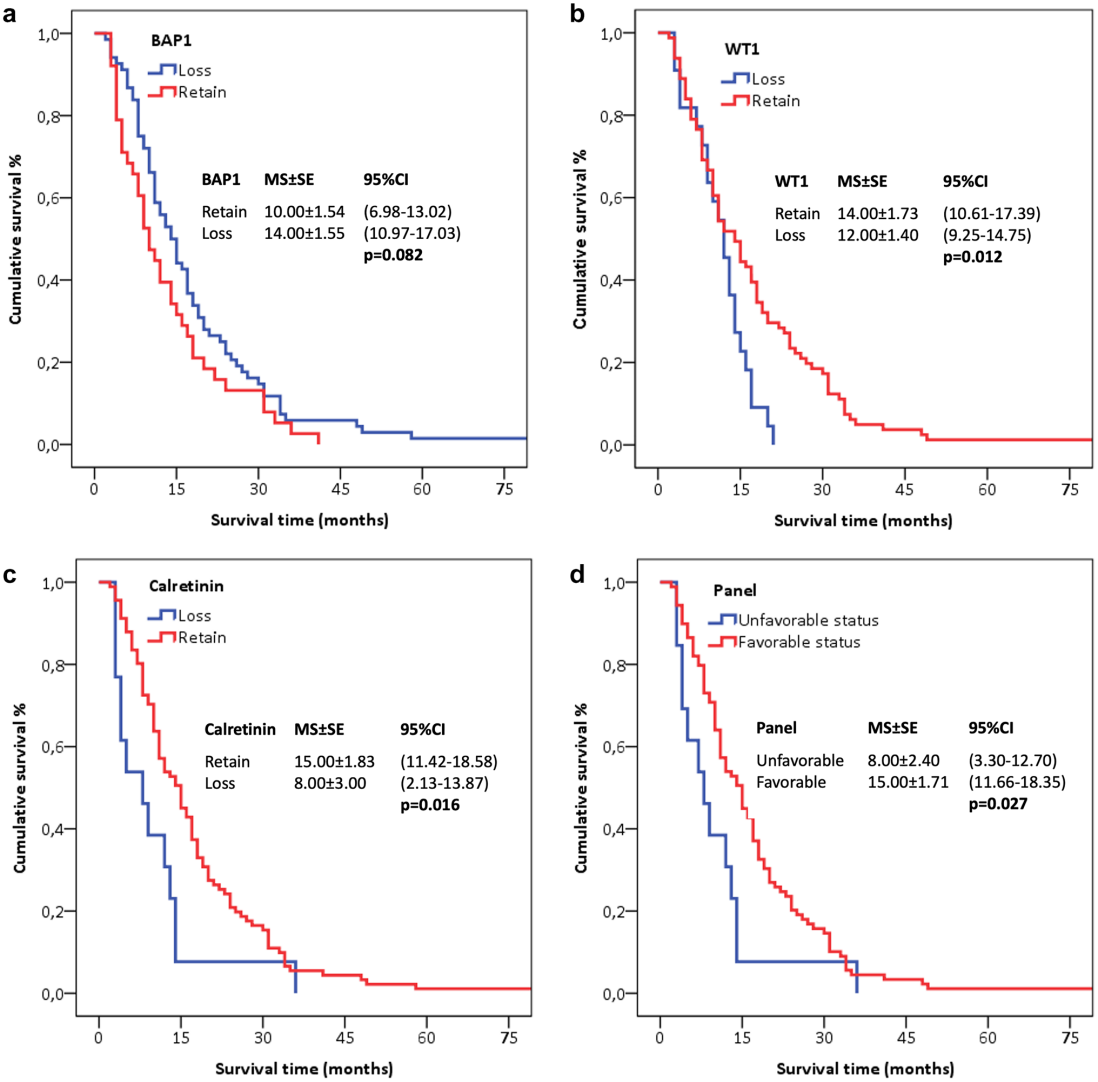
Survival curves of patients according to their immunohistochemical staining results and panels. Unfavorable status: In the presence of BAP1 expression, one or both of the other two markers, or loss of expression of all three markers. Favorable status: In the presence of all three markers or loss of BAP1 expression and expression of one or two other markers

Survival time was longer in patients with BAP-1 loss, but not statistically significant (*p* = 0.082). Survival time of patients with WT1 and calretinin expression was longer than that of patients with loss of WT1 expression (*p* = 0.012 and *p* = 0.016, respectively). Median survival time was longer in patients with favorable status (15 ± 1.7 months) than in patients with unfavorable status (8.0 ± 2.4 months) (*p* = 0.027) (Fig. 3, Table 3).

**Table 3:** Results of univariate/multivariate analysis of patient survival probability by immunohistochemical staining results and panels

Variables	*HR	p	**HR	*p*
Age				
< 65	1			
> 65	1.40 (0.94–2.08)	0.098		
Sex				
Male	1			
Female	0.80 (0.53–1.20)	0.276		
Histopathology				
Epitheloid	1			
Non-epitheloid	2.18 (1.02–4.67)	**0.045**		
Stages				
I–II–IIIA	1			
IIIB–IV	2.08 (1.38–3.15)	**0.001**		
Response				
Progressive	1			
Stable disease	0.34 (0.21–0.55)	** < 0.001**		
Objective response	0.22 (0.13–0.39)	** < 0.001**		
BAP-1				
Retain	1		1	
Loss	0.71 (0.47–1.06)	0.095	0.54 (0.35–0.83)	**0.004**
WT1				
Retain	1		1	
Loss	1.85 (1.12–3.04)	**0.016**	1.75 (1.06–2.90)	**0.029**
Calretinin				
Retain	1		1	
Loss	1.99 (1.01–3.59)	**0.023**	2.09 (1.14–3.84)	**0.017**
Panels				
Unfavorable status	1		1	
Favorable status	0.53 (0.29–0.96)	**0.036**	0.50 (0.27–0.92)	**0.026**

Bold *p*-values show statistical significance

*HR* Hazard ratio

^*^Univariate analysis

^**^Adjusted probability with histopathology and stage

In univariate analysis, HR was higher in patients with loss of WT1 and calretinin expression compared with WT1 and calretinin expression, non-epithelioid type compared with epithelioid type, advanced stage compared with early stage, progressive disease to chemotherapy compared with stable or objective response. However, there was no significant difference in loss of BAP1 expression compared to those expressing BAP1; risk reduction was observed (*p* = 0.095). After adjustment for histopathology and stage, HR was significantly lower in cases with loss of BAP1 expression or WT1 and calretinin expression. Considering IHC staining results, HR was lower in patients with “favorable status” than in those with “unfavorable status” in multivariate analysis.

## Discussion

This study investigated the role of IHC staining of BAP1, WT1, and calretinin and panels of their combinations in mesothelioma tumor tissue samples in predicting prognosis and response to chemotherapy in PM patients. This is to our knowledge, the first study to show a panel of immunohistochemical markers that can stratify mesothelioma patients with favourable and unfavourable profile to predict response and survival after first-line pemetrexed and platinumin. After adjustment for histopathology and stage, loss of BAP1 expression and the presence of WT1 and calretinin expression were associated with improved survival.

### Single biomarkers and prognosis

BAP1, WT1, and calretinin are useful biomarkers for histopathologic diagnosis of PM (Louw et al. 2022). There are few studies showing that the expression of WT1 and calretinin indicates a good prognosis in PM (Cedrés et al. 2014; Kao et al. 2011), while BAP1 has been the subject of numerous studies (Louw et al. 2022; Cigognetti et al. 2015; Farzin et al. 2015; Pulford et al. 2017). Loss of BAP1 expression in patients treated with cisplatin/pemetrexed was found to be associated with longer survival after chemotherapy, suggesting that loss of BAP1 expression may have predictive value for this chemotherapy regimen (Louw et al. 2022; Farzin et al. 2015). Inversely BAP1 expression was found to increase the effect of gemcitabine in mesothelioma cell lines, showing that the expression of BAP1 may affect outcomes differently according to type of chemotherapy (Guazzelli et al. 2019).

Loss of BAP1 is seen in 50–70% of PM patients (Carbone et al. 2012, 2020; Nasu et al. 2015). In 64.5% of patients in the present study, loss of nuclear staining was detected in tumor tissue, indicating the presence of a BAP1 mutation by IHC staining. The loss of BAP1 expression was higher in the female patients, diverging from other studies (Kindler et al. 2018; McGregor et al. 2015). In this cohort, the loss of BAP1 expression by histopathologic subtype was not significantly different in line with previous studies (Louw et al. 2022; Chapel et al. 2020; Cigognetti et al. 2015; Farzin et al. 2015).

The median survival was 14.0 months in patients with loss of BAP1 expression and 10.0 months in patients with retained expression in this study, but the difference did not reach statistical significance (*p* = 0.087). However, after adjusting for histopathological type and stage, loss of BAP1 expression was associated with a good prognosis. In the only validation study on BAP1 and survival after first-line platinum pemetrexed, in two different centers in Denmark and Australia, the median survival time in the Danish patient group was 14.2 months in patients with loss of BAP1 expression versus 6.5 months in patients with retained BAP1 expression and 18.9 versus 10.8 months in the Australian group, respectively (*p* < 0.001) (Louw et al. 2022). It was reported that loss of BAP1 expression was associated with better survival in patients in both cohorts (Louw et al. 2022). In other studies, median survival ranged from 13.1 to 16.1 months in patients with BAP1 loss and from 6.3 to 6.9 months in patients with retained BAP1expression (Cigognetti et al. 2015; Pulford et al. 2017; McGregor et al. 2017).

In this study, we have shown that loss of BAP1 expression is associated with a good prognosis regardless of histopathology. The results of other studies on the impact of BAP1 loss on survival by histopathologic subtype in PM are conflicting (Cigognetti et al. 2015; Farzin et al. 2015; Pulford et al. 2017; McGregor et al. 2015, 2017; Cantini et al. 2020; Dudnik et al. 2021). The low incidence of sarcomatous subtype is likely a confounding factor in the general patient population. Therefore, the efficacy of loss of BAP1 expression as a prognostic marker could be more clearly discussed in larger, multicenter studies including patient groups with sufficient subtype distribution.

WT1 nuclear staining positivity for PM is reported to have a sensitivity of 70–95% (Scattone et al. 2012). WT1 is not presented in lung adenocarcinomas or squamous cell carcinomas; thus, it is useful in differentiating these malignancies from epithelioid mesothelioma. WT1 sensitivity has been reported to be approximately 10–45% in sarcomatoid PM (Louw et al. 2022; Scattone et al. 2012). In this study, 78.8% of patients were positive for WT1 expression. WT1 expression was higher in the epithelioid subtype (*p* < 0.001). The National Cancer Institute Cancer Panel recently identified WT1 as a major tumor-associated antigen that could be targeted (Cheever et al. 2009). One possible explanation for the better survival of patients with WT1 expression is the immune response observed in WT1-expressing tumors. WT1 shows tumor-specific overexpression and the ability to elicit active and humoral immunity. In a preclinical study using cell lines expressing WT1, WT1 vaccine peptides induced CD4- and cytotoxic CD8 WT1-specific T-cell responses (May et al. 2007). Since WT1 is highly expressed in PM and elicits an immune response, it is an attractive target for immunomodulatory PM therapy. In a pilot study of nine patients vaccinated with a vaccine composed of analogous WT1 peptides, the vaccine proved safe and elicited an immune response (Krug et al. 2011). Two ongoing phase 2 trials are evaluating an adjuvant WT1 analog peptide vaccine after completion of combination therapy for PM (Zauderer et al. 2017; Eguchi et al. 2017).

In a study examining the relationship between WT1 expression and PM prognosis, WT1 expression was 78.1% (Cedrés et al. 2014). Median survival time in cases expressing WT1 was 16.4 months, versus 2.3 months in patients without WT1 expression. The difference in median survival was also present after multivariate analysis to which histologic type was added (Cedrés et al. 2014). Pezzuto et al. studied the deletions of ki67, WT1, and p16 in 45 patients with peritoneal mesothelioma. Median survival was significantly higher in patients with expressed WT1 (29.2 months) than in patients without WT1 expression (5.0 months) (Pezzuto et al. 2020). Similar results were found in a study on peritoneal mesothelioma (Husain et al. 2018).

In our cohort, WT1 expression was associated with longer survival both in univariate and in multivariate analysis. Median survival was 14 months in patients with WT1 expression but only 12 months without expression. The results of this study indicate that WT1 may also be an prognostic marker for PM.

Calretinin expression is detected by IHC staining with a sensitivity of 80–100% in epithelioid and 50–60% in sarcomatoid subtype (Louw et al. 2022). Since the positivity of calretinin in lung adenocarcinomas and renal cell carcinomas has been reported at a low rate of 0–10% in various publications, it is a useful marker in the differentiation of lung adenocarcinomas and renal cell carcinomas (Louw et al. 2022).

In this study group, calretinin expression was detected by IHC staining in 87.6% of patients. Calretinin expression was present in 93.2% of the cases in the epithelioid subtype and 16.2% in the sarcomatoid subtype (*p* < 0.001).

Kao et al. performed the first study to investigate the association between calretinin expression and prognosis in PM (Kao et al. 2011). The survival analysis was performed using calretinin and peripheral blood neutrophil-to-lymphocyte ratio in 85 PM patients who underwent extrapleural pneumonectomy. Median survival was 35.8 months in patients with 67–100% calretinin expression in tumor tissue samples, 14.5 months in patients with 34–67%, and 6.9 months in patients between 0 and 33%. Multivariate analysis showed that the group with the lowest calretinin percentage had a worse survival probability (Kao et al. 2011). Thapa et al. analyzed the association between calretinin and caveolin-1 expression and survival in 329 PM patients. In a multivariate analysis, they found that calretinin expression was associated with longer survival (Thapa et al. 2016).

In a review of factors associated with survival in 910 PM patients in South Wales, the median survival was 10.9 months in patients with calretinin expression and 5.5 months in patients without calretinin expression. In multivariate analysis, patients without calretinin expression were found to have a twofold higher risk of death (Linton et al. 2014). The study by Cedres et al. was one of the few studies that investigated the relationship between calretinin expression and PM prognosis. Calretinin was expressed in 41 of 47 (83.7%) patients. While the median survival time was 16.6 months in patients with calretinin expression and 5 months in patients without expression, the survival benefit was not statistically significant (Cedrés et al. 2014). The small number of cases might affect the reliability of this study.

The patients in this study had better survival in the calretinin-expressing group. The median survival time was 15 months in patients with calretinin expression and 8 months in patients without expression (*p* = 0.016). Calretinin expression was also associated with a good prognosis in multivariate analysis.

There is lack of information on the possible mechanisms of the relationship between the presence of calretinin expression and prognosis, similarly with WT1. Recent data suggest that calretinin plays a role in maintaining mesothelial cell viability and proliferation in vitro and that downregulation occurs when apoptosis pathways are activated (Blum and Schwaller 2013). Calretinin expression may also be a surrogate marker for tumor differentiation. Takeshima et al. observed higher calretinin scores in well-differentiated tumors with a more favorable prognosis (Takeshima et al. 2010).

### Single biomarkers and response

In this study, we also investigated whether the expression of BAP1, WT1, and calretinin is useful for predicting response to platinum pemetrexed treatment. We have shown that expression of BAP1 cannot predict response to chemotherapy, but expression of WT1 or calretinin may have a predictive value. Although WT1 and calretinin can predict response to chemotherapy, they were also highly expressed in patients with progressive disease, thus clinical implementation is hampered.

### Biomarker panels, unfavourable and favourable

The predictive value of the biomarkers increase by the use of combination panels. We observed the presence of BAP1 expression, one or both of the other two markers, or loss of expression of all three markers was designated “unfavourable status”, where most patients progressed and none had a response. Opposite, we designated “favourable status” with the presence of all three markers or loss of BAP1 expression and expression of one or two other markers where approximately 1/3 of patients had response, 1/3 had stable disease and 1/3 had progressive disease. The rate of progressive response to chemotherapy is significantly higher in patients with unfavorable status than in favorable status (84.6% versus 36.7%). The rate of progressive disease in patients with unfavorable status was similar in both the epithelioid/biphasic and sarcomatoid subtypes. In addition, the median survival of patients with favorable status was longer than that of patients with unfavorable status [15 months versus 7 months, HR 0.50 (0.29–0.96) 95%CI] (Fig. 3 and Table 3) and stratified the long versus short survivors better than the single biomarkers.

The importance of these findings, besides stratification according to survival, is that there is an apparent subgroup of BAP1 loss patients that will not respond to chemotherapy at all, given that both WT1 and calretinin both are negative, a “triple-negative” subtype of mesothelioma (5/5 patients). Equally bad is the case where BAP1 is positive but with both WT1 and calretinin negative, where all patients progressed (5/5 patients). Interestingly, when both WT1 and calretinin are positive, the BAP1 status seems not to play a role for the outcome, with equal chance of response, stable or progressive disease.

This study has both strengths and limitations. The strengths include that the study group is a well followed, relatively homogeneous group in terms of demographic characteristics and asbestos exposure. All patients were exposed to environmental asbestos in rural areas. In this population, the ratio of female is higher and the disease occurs at a younger age than in the occupational group. These characteristics may have an impact on treatment success. The fact that all patients included in the study were treated with the same chemotherapy regimen and with chemotherapy alone is an advantage of the study. In addition to BAP1, WT1 and calretinin, which are routinely used to diagnose MPM, have also been shown to influence the prognosis of PM and response to chemotherapy. A panel was assembled from these three markers.

The limitations include single-center setup and the limited number of patients with sarcomatoid subtypes.

In conclusion, this study shows promising results suggesting that BAP1, WT1 and calretinin, simple IHC biomarkers used in the routine pathology lab, may be useful in determining response and prognosis for PM after platinum pemetrexed chemotherapy. These results remain to be validated in larger studies.

## Data Availability

The data that support the findings of this study are available from the corresponding author upon reasonable request.
